# Effect of memantine on post-operative cognitive dysfunction after cardiac surgeries: a randomized clinical trial

**DOI:** 10.1186/s40199-017-0190-0

**Published:** 2017-11-21

**Authors:** Saba Ghaffary, Padideh Ghaeli, Azita Hajhossein Talasaz, Abbasali Karimi, Maryam Noroozian, Abbas Salehiomran, Arash Jalali

**Affiliations:** 1 Hematology and Oncology Research Center, Tabriz University of Medical Sciences, Tabriz, Iran; 2 Departments of Pharmacotherapy, Faculty of Pharmacy, Tehran University of Medical Sciences, Tehran, Iran; 3 Tehran Heart Center, Tehran University of Medical Sciences, Tehran, Iran; 4 Memory and Behavioral Neurology Department, Roozbeh Hospital, Tehran University of Medical Sciences, Tehran, Iran

**Keywords:** Post-operative cognition dysfunction (POCD), Glutamate, Wechsler memory test (WMT), Memantine, Cardiac surgeries

## Abstract

**Background:**

Post-operative cognitive dysfunction (POCD) is an important complication of cardiac surgeries. Glutamate plays a critical role in physiologic and pathologic conditions in the brain. Due to the role of glutamate in ischemia, this study is designed to identify the effect of memantine in prevention of POCD early and late after cardiac surgeries.

**Methods:**

In this randomized clinical trial, 172 patients with ages 45–75 years old who underwent elective cardiac surgery were enrolled. For patients in memantine group, 5 mg of memantine per day administered at least 48 h before surgery and increased to 10 mg per day during the first 24 h after surgery and continued for 3 months. A brief Wechsler memory test (WMT) was administered before, three to 5 days after, and 3 months after surgery for both groups.

**Results:**

Both groups demonstrate standard pattern of cognitive dysfunction after surgery and in follow up. Pre- and post-operative WMT score showed significant improvement in memantine compared to control group (*P* < 0.001) both in unadjusted and adjusted with confounding factor analysis. Unadjusted pre-, post-operative, and follow up WMT score improved significantly after 3 months in memantine group (*P* = 0.006).

**Conclusion:**

Pre-operative administration of memantine protects patients from POCD following cardiac surgeries. In addition, it improves cognitive function 3 months after surgery.

**Trial registration:**

The trial was registered in the Iranian Registry of Clinical Trials (registration number: IRCT201303168698N12).

**Graphical abstract:**

Memantin effect on POCD.
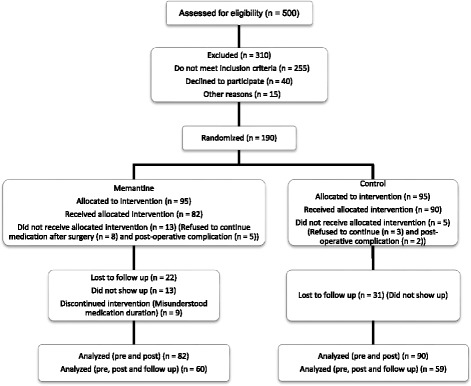

## Background

Post-operative cognition dysfunction (POCD), including deficits of memory, concentration and psychomotor speed is one of the neurologic complications after cardiac surgeries [1]. Different theories have been suggested for intraoperative mechanisms and pathophysiology of POCD. The formation of cerebral microemboli, Inflammation, anesthesia, hyperglycemia, oxygenation, speed of cardiopulmonary bypass rewarming temperature, hypoxia after surgery, duration of surgery, alterations in the cerebral perfusion and temperature change are some mechanisms suggested for POCD [2]. The cognitive dysfunction incidence is highest at discharge (50 to 80%) and is reduced to 10 to 30% in 6 months [3]. Although cognitive performance improves a year after cardiac surgery, it starts to decline from 1 to 5 years and reaches to 42% [4]. The exact pathophysiology of neurological complications after cardiac surgeries has not been clearly determined [5].

Glutamate plays a major role in many neurologic functions. Its level rise 50 fold the normal value within a short time in acute injury and lead to cell death following injury [6]. In addition, cells are vulnerable to physiologic concentrations of glutamate in pathologic conditions [7]. It should be mentioned that the normal function of N-methyl-D-aspartate (NMDA) receptor is critical in learning and memory formation. Subsequently, it is very important to preserve normal physiologic activity of NMDA receptors in order to preserve the normal neurological functions and avoid clinical side effects [8].

Memantine is the first uncompetitive, low-to-moderate affinity antagonist of NMDA receptor. Because of its affinity for the receptor, it is not leading to learning impairment or psycho-mimetic dysfunction like ketamine. Based on the critical role of glutamate in acute brain injury, and proven effect of memantine on memory we hypothesized that memantine may have a protective effect on POCD.

## Methods

### Patient enrollment

After institutional review board approval 172 patients (82 in memantine and 90 in control groups), who were undergoing elective cardiac surgery, assigned to this open-label randomized clinical trial. Participants younger than 45 and older than 75, and patients with a history of symptomatic cerebrovascular disease, stroke, seizure, psychiatric illness, active liver disease, or inability to calculate (due to perform Wechsler test) were excluded from enrollment. All surgeries were performed on-pump and operated by the same medical team under the same conditions. Simple randomization was conducted in this study by using computer-generated random sequence.

### Measurement of Neurocognitive function

A brief neurocognitive test battery (Wechsler Memory Test) was administered before surgery and three to 5 days after surgery for both memantine and control groups. Wechsler Memory Test (WMT) is a memory assessment test which composed of seven different parts to assess the different parts of memory [9]. Assessments were performed individually by the same researcher (Trained resident of Pharmacotherapy). The assessment was performed without any interruption and in a quiet place for all patients before and after surgery.

WMT evaluates the followings: 1) Asking six questions to distinguish a patient who has aphasia or senile dementia; 2) Asking five questions regarding orientation to time and place; 3) Ability of mind control by special digits pattern; 4) Ability of subjects to recall the details of read text; 5) Ability to count separate digits in forward and reverse orders; 6) Dozens of words with or without any association in meaning was read to patients to assess ability to remember the association between words; 7) Assess the ability of subjects to reproduce a series of geometric shapes after a 10-s exposure [9].

The sum of scores from different parts of Wechsler memory test is added with the age constant. Then the new score is adjusted with respect to the standard Wechsler memory test table to reach the final score.

### Intervention

For patients who randomly allocated to memantine group, 5 mg of memantine per day administered at least 48 h before surgery and increased to 10 mg per day (5 mg bid) during first 24 h after surgeries. Memantine 10 mg/day continued for patients until 3 months after discharge. Memantine was tolerated well and no patient reports any cardiac or non-cardiac side effects during administration. Memantine does not have any interaction with all administered medications and procedures during hospitalization for cardiac surgeries and did not change any cardiac parameters.

### Standard protocol approval, registration and patient consents

The protocol of the present study was approved by the local Ethics Committee of Tehran Heart Center, Tehran University of Medical Sciences, Iran on 12 March 2013. The trial was registered in the Iranian Registry of Clinical Trials (registry number: IRCT201303168698N12). All patients were informed about the trial and gave a written informed consent before the study initiation.

### Follow up

We had 3 months period of follow up for all patients in both memantine and control groups. After 3 months from discharge, patients were referred to the risk factor follow up clinic. Patients cognitive functions were evaluated by the same Wechsler memory test in both groups. All tests were performed in a quiet room without any interruption. Patients who were administered 10 mg memantine were asked about the adherence to the medication. Patients who did not continue memantine after discharge and could not complete the 3 months of medication period excluded from follow up.

### Statistical analysis

Continuous variables were described with mean and standard deviation (SD) or median with 25^th^ and 75^th^ quartiles whenever the variables did not normally distributed. They were compared between intervention groups using independent samples t-test or Mann-Whitney U-test. Categorical variables were expressed with frequency and percentage, and were compared between memantine and control groups applying chi-squared or Fisher’s exact test. Post-operative WMT score and WMT score change were compared between intervention groups adjusting for confounder variables using multiple linear regression models. WMT score change for patients who were followed up 3 months after surgery were analyzed applying repeated measures analysis of variance (ANOVA). *p* values less than or equal to 0.05 were considered statistically significant. Statistical analysis was performed using IBM SPSS statistics for Windows, version 20.0.

## Results

**Fig. 1:**
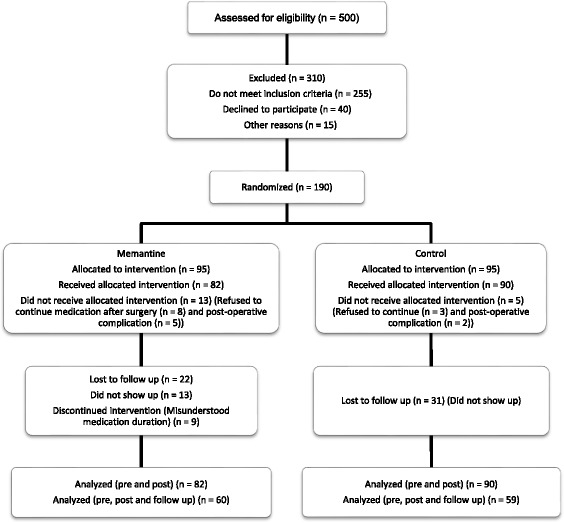
Screening, randomization and follow up of study participants

In this study, 500 patients were screened for eligibility from which 255 did not meet the inclusion criteria, 40 refused to be included in the trial and 15 excluded because of other reasons. Finally, 190 patients were randomized in two groups. There were 18 dropouts during the study, that is, 13 in memantine group and five in the control group. Therefore, 172 patients, 82 in the memantine and 90 in the control group, completed the period of hospitalization part of the study. From 172 patients, 22 and 31 individuals did not show up for follow up in memantine and control groups, respectively (Fig. 1).

From 172 patients 145 were isolated coronary artery bypass graft (CABG) (84.3%), 14 were CABG plus valve replacement (8.1%), six patients were CABG plus other type of surgery (3.5%), and seven were valve or other type of surgery (4.1%).

**Fig. 2:**
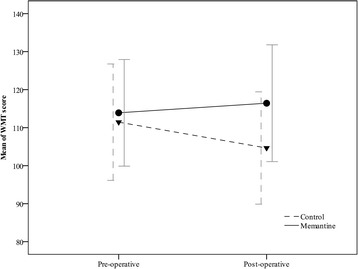
Pre- and post-operative WMT score in all patients

The corresponding pre- and post-operative data were included in the final analysis. Our analysis shows that WMT score improves after 3 months in both groups (memantine and control) with different trend of improvement during this 3 months (*p* = 0.02; Fig. 2). From baseline parameters, being smoker, dyslipidemia, pre-operative antiplatelet consumption, blood transfusion, LDH level, cross clamp time and perfusion time were significantly different between groups and known as confounding factors (*p* < 0.05; Tables 1 and 2).

**Table 1:** Demographic and clinical characteristics of patients (Categorical data)

Parameters		Control *n* = 90(%)	Memantine *n* = 82(%)	p value
Sex	Female	10(11.1)	9(10.9)	0.586
	Male	80(88.9)	73(89.0)	0.586
Risk Factors*	Current Smoker	12/89(13.5)	26(31.7)	0.004
	Alcohol Consumption	15 (16.7)	19(23.2)	0.190
	Diabetes	30(33.3)	30(36.6)	0.387
	Dyslipidemia	46(51.1)	56(68.3)	0.016
	Hypertension	45(50.0)	44(53.7)	0.372
	Positive Family History	40(44.4)	30(36.6)	0.186
	Myocardial Infarct	23(25.6)	33(40.2)	0.260
	CHF	4(4.4)	7(8.5)	0.217
Pre-operative Medication*	β-Blocker	73/87(83.9)	72/78(92.3)	0.780
	CCB	13/87(14.9)	9/78(11.5)	0.341
	Nitrates	75/87(86.2)	69/78(88.5)	0.422
	Diuretics	21/87(24.1)	20/78(25.6)	0.482
	ACEI	31/87(35.6)	29/78(37.2)	0.482
	ARB	31/87(35.6)	25/78(32.1)	0.375
	Anticoagulation	21/87(24.1)	25/78(32.1)	0.169
	Digoxin^⁑^	5/87(5.7)	5/78(6.4)	0.556
	Amiodarone^⁑^	2/87(2.3)	1/78(1.3)	0.541
	Oral antihyperglycemic Agent	14/88(15.9)	16/78(20.5)	0.285
	Insulin	14/88(15.9)	13/78(16.7)	0.530
	Bronchodilator	11/88(12.5)	6/78(7.7)	0.224
	PPI	7/17(41.2)	17/57(29.8)	0.276
	Antiplatelet	69/87(79.3)	51/78(65.4)	0.034
Complication*	Blood Transfusion	47/87(54.0)	27/75(36.0)	0.016

* *p* values are generated by Chi-squared test

^⁑^ *p* values are generated by Fisher’s exact test

Categorical variables are presented in percent change (%). *CHF* chronic heart failure, *CCB* calcium channel blocker, *ACEI* angiotensin converting enzyme inhibitor, *ARB* angiotensin receptor blocker, *PPI* proton pump inhibitor

**Table 2:** Demographic and clinical characteristics of patients (Continuous data)

Parameters^a,b^	Control	Memantine	p value
Age	56.59 ± 6.71	56.39 ± 7.15	0.277
BMI (kg/m2)	26.76 ± 3.37	26.98 ± 4.29	0.250
HDL (mg/dL)	35.82 ± 8.15	36.25 ± 11.25	0.174
LDL (mg/dL)	90.30 ± 32.50	93.14 ± 33.52	0.755
LDH (IU/L)	245.53 ± 88.32	185.18 ± 59.76	<0.001
EF	46.69 ± 8.35	44.46 ± 9.50	0.339
Total Graft	3.42 ± 0.98	3.52 ± 1.03	0.841
Pre-operative WMT score	111.44 ± 15.30	113.90 ± 14.03	0.323
FBS (mg/dL)	96 (85–117)	96.5 (89–117)	0.557
Cholesterol (mg/dL)	146 (113.7–169.5)	146 (118–189)	0.447
Triglycerides (mg/dL)	134.5 (92.7–166.2)	124 (101–186)	0.530
Serum Creatinine (mg/dL)	0.9 (0.8–1)	0.8 (0.8–1)	0.425
Cross Clamp Time (Minute)	41 (30–50)	45 (40–59)	0.002
Perfusion Time (Minute)	70 (55–90)	80 (66.2–100.8)	0.005

Continuous variables are described in mean ± standard deviation (SD) and median ± range

^a^Parametric variables are analyzed with Student t test

^b^Non-parametric variables are analyzed with Mann whitney u test. *BMI* body mass index, *HDL* high-density lipoprotein, *LDL* low-density lipoprotein, *LDH* lactate dehydrogenase, *EF* ejection fraction, *WMT* Wechsler memory test, *FBS* fasting blood sugar

**Fig. 3:**
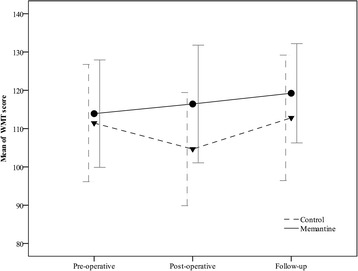
Trend of pre- and post-operative and after 3 months WMT scores in patients who followed up

The baseline levels of cognition were in the same range and did not differ statistically between groups (*p* = 0.275). The mean value of pre-operative WMT score in control group decreases from 111.44 (±15.31) to 104.64 (±14.77) whereas it increases from 113.90 (±14.03) to 116.42 (±15.36) in memantine group (*p* < 0.001). In addition, the change of WMT score mean pre- and post-operatively showed improvement in memantine group (2.5 score), while this change is negative in control group (−6.8 score with *p* < 0.001). Furthermore, after adjustment with confounding factors WMT score was significantly higher in memantine group (p < 0.001). Unadjusted effect of memantine administration 3 months after surgery significantly improved the WMT score (*p* = 0.006). Whereas, adjusted data with confounding factors did not show any significant effect after 3 months. The trend of pre- and post-operative and follow up WMT scores in patients who do not miss in follow up are illustrated in Fig. 3. In addition, the scores for the various memory domains are presented in Table 3.

**Table 3:** WMT Scores for the various memory domains

Group	Personal and current information	Orientation	Mind control	Logical memory	Memory span	Associate learning	Illustration reproduction	Total	Corrected score with age
Baseline Test	5.62650	4.975904	8.795181	6.180723	11.31325	12.27711	10.44578	59.49398	113.5663
Control	5.80198	4.980198	8.158416	5.435644	9.98019	12.16832	9.62376	56.17822	108.1980
After Test	5.65060	4.987952	8.795181	6.843373	10.51807	13.44578	10.73494	60.95181	116.1446
Control	5.77227	4.970297	7.891089	5.366337	8.36633	11.13861	9.24752	52.65347	101.8119
Follow up Test	5.71428	5.053571	8.964286	4.964286	9.94642	15.41071	11.98214	61.85455	118.5536
Control	5.81818	4.984848	7.984848	4.075758	9.66667	14	10.51515	57.75758	109.6970

## Discussion

Prevention of POCD is very important due to its extensive effect on different aspects of patient life after surgery. Memantine is not only an antagonist of NMDA but also plays a neuroprotective role against neuronal degeneration of amyloid beta (Aβ) [10]. Memantine is a safe medication and does not have any caution in patients with cardiovascular disease. Subsequently, it was chosen as a novel medication before cardiac surgery to protect patients from POCD. In this study, both control and memantine groups had the same range of cognitive function at baseline before surgery. However, our results show that administration of 5 mg/day of memantine at least 48 h before surgery, and an increased dose in 24 h after surgery to 10 mg/day, decreases the POCD significantly in comparison to the control group. In addition, it is demonstrated that patients who received 10 mg/day of memantine after surgery for 3 months, experience a better cognitive function in with higher score of WMT after 3 months (unadjusted). It seems that the high number of patients lost to follow up is the reason for insignificant results in adjusted analysis with confounding factors.

In previous studies, other N-methyl-D-aspartate (NMDA) antagonists role on cognitive dysfunction after surgery and traumatic brain injury (TBI) have been studied by several researchers. In a double-blind, placebo-controlled, crossover trial by Meythaler et al., amantadine was administered within the first 24 h to the patients who had a TBI with a Glasgow Coma Scale score of 10 or less. In their study patients divided into two groups. Group 1 received amantadine the first 6 weeks after injury and then placebo for the second 6 weeks, whereas group 2 received placebo and amantadine in the first and second 6 weeks, respectively. Their results show that cognitive function improves more rapidly in patients in group 1 who had started on amantadine in the first 3 months after TBI [11].

The effect of ketamine during anesthetic induction was compared with normal saline in cardiac surgeries [12]. The data showed that patients who received ketamine in anesthesia induction had better cognitive dysfunction, 1 week after cardiac surgery [12]. In contrast, another study which was performed in elderly patients after orthopedic surgeries, did not support the advantage of ketamine administration during anesthetic induction on POCD [13].

High level of extracellular concentration of glutamate is present in the rodent brain and spinal cord during ischemia or trauma [14, 15]. Previous research confirmed that high concentrations of glutamate, 100–500 mM, lead to cell death via activation of NMDA receptors [6]. In spite of potential toxic effect of high level of glutamate, its activity in a physiological concentration is absolutely required for normal brain function and long term improvement of learning and memory. Blockade of NMDA receptors leads to apoptosis in the developing brain [16]. Subsequently, complete blockade of NMDA receptors can cause serious complications such as coma, drowsiness, and hallucinations [17]. Based on the critical role of glutamate, its excitotoxicity effect should be blocked without interfering with its physiological action.

Although the concentration of glutamate increases to 10–100 fold of normal level after ischemia and brain injury, it remains high for 10–30 min and remains slightly high over days to weeks [18, 19]. Ikonomidou et al. suggested that such mild elevation of glutamate concentration may display a self-defense mechanism of the injured brain, which may improve survival of endangered neurons and facilitate tissue repair [20]. Subsequently, NMDA antagonist administration early after TBI (1–7 h after trauma) can harm neurons [21]. However, NMDA antagonist can play a protective role if it is administered prior to traumatic injury. Based on these findings, although glutamate kills neurons immediately after injury, it starts to assist brain repair shortly thereafter and preserves endangered neurons in long term. According to the research done by Ikonomidou et al., NMDA antagonists are not suitable neuroprotective drugs for use in human emergency medicine. The neuroprotective effect of NMDA antagonists is only in a situation that administered before injury and for a very short period (minutes) after that [20].

NMDA channels are open on average for only several milliseconds during normal synaptic activity and memantine cannot accumulate in the channels. Accordingly, synaptic activity continues unabated in the presence of memantine in physiologic condition. Fixed concentration of memantine blocked more NMDA receptors activity when the concentration of glutamate increased to pathologic levels. As a matter of fact, memantine cannot block NMDA receptor activity in normal neurological function but it is effective on blockade of receptor at higher concentrations. Subsequently, memantine becomes a very effective NMDA receptor blocker under excitotoxic conditions and prolonged activation of NMDA receptors [8].

Due to the critical effect of memantine on NMDA receptors in physiologic and pathologic conditions, it is reasonable to see the positive neuroprotection during cardiac surgeries. The pre-operative administration of memantine can protect neurons from excessive amount of glutamate released during cardiopulmonary bypass, whereas it does not interfere with normal level of glutamate function [22].

In addition, our study demonstrates strong positive effect on patient’s cognitive function in long term. Patients are in a high risk of vascular dementia after cardiac surgeries. In chronic disorders, the NMDA receptors become hyperactive for longer periods of time than occur during normal neurotransmission with a low level of glutamate. It is obvious that in neuronal damage in chronic disorders the elevation of extracellular glutamate is not necessary to induce an excitotoxic mechanism. In this situation NMDA receptors activity is increased because of relief from normal Mg^2+^ Blockade. These NMDA receptors over activity lead to neuronal injury or apoptotic-like cell death. Accordingly, memantine can affect NMDA receptors over activity in the presence of low glutamate concentration [23]. In parallel with the mentioned mechanism in this study, patients who received memantine for 3 months after surgery showed significant improvement in their cognitive function.

Moreover, previous studies showed that Memantine rescues transient cognitive impairment induced by High-Molecular-Weight Aβ Oligomers in mice [24]. Hence, effect of memantine on POCD is justifiable with the blockade of glutamate response on NMDA channel by blocking excessive calcium influx into the cell and reduction of Aβ formation in neurons [25].

## Conclusion

Protective effect of memantine on POCD can be presented by its selective affinity to NMDA channel in different concentration of glutamate. Memantine blocks the NMDA channel during ischemia and early after that. Consequently, memantine protects brain from toxic effect of glutamate’s high concentration. Whereas, memantine maintains the normal action of glutamate in physiologic concentration and does not interrupt brain learning function.

It is hypothesized that pre-operative administration of memantine protects patients from early post-operative cognitive dysfunction in patients who undergo cardiac surgeries. Memantine does not have any drug interaction and patients did not contribute to any adverse effects when receive memantine. In conclusion, memantine is a safe neuroprotective agent against POCD after cardiac surgeries and it is tolerated well by patients pre- and post-operatively.

### Study limitations

Due to the transient high concentration of glutamate after acute injury, it might be promising to design a study with pre-operative single high-dose of memantine. Because of financial limitations, paraclinic imaging has not been included in this study. For future studies paraclinic imaging, before and after surgery, is recommended in order to better provide brain changes comparison after cardiac surgeries. In this study, neither patients nor the personnel who performed tests were blind. It is worth mentioning that patients did not receive any placebo. Subsequently, double-blind, placebo-controlled studies are recommended.

## Abbreviations

ANOVA: Applying repeated measures analysis of variance
Aβ: Amyloid beta
CABG: Coronary artery bypass graft
NMDA: N-methyl-D-aspartate
POCD: Post-operative cognition dysfunction
SD: Standard deviation
TBI: Traumatic brain injury
WMT: Wechsler memory test
